# Exploring the competencies of operating room nurses in mobile surgical teams based on the Onion Model: a qualitative study

**DOI:** 10.1186/s12912-023-01417-3

**Published:** 2023-08-02

**Authors:** Aifang Niu, Huijuan Ma, Zhe Chen, Xiaoli Zhu, Yu Luo

**Affiliations:** 1 School of Nursing, Third Military University / Army Medical University, No. 30 Gaotanyan Street, Shapingba District, Chongqing, P.R. China; 2 Army Health Service Training Base, Third Military University / Army Medical University, No. 30 Gaotanyan Street, Shapingba District, Chongqing, P.R. China; 3 Emergency department, General hospital of xinjiang military command, No. 754 Beijing Street, Urumqi, Xin Jiang P.R. China

**Keywords:** Competency, Operating room nurse, Mobile, Surgical team, Qualitative study

## Abstract

**Background:**

With the frequent occurrence of public health emergencies, conflicts and natural disasters around the world, mobile surgical teams are becoming more crucial. The competency of the operating room (OR) nurse has a substantial impact on the effectiveness and quality of the surgical team’s treatment, still there is limited knowledge about OR nurse competencies in mobile surgical teams. This study aimed to explore the competencies of OR nurses in mobile surgical teams based on the Onion Model.

**Methods:**

We conducted a qualitative descriptive study of participants from 10 mobile surgical teams in 2022. Twenty-one surgical team members were interviewed, including 15 OR nurses, four surgeons, and two anesthesiologists. Data were collected through semi-structured interviews. The data were analyzed using Mayring’s content analysis.

**Results:**

Twenty-eight competencies were found in the data analysis, which were grouped into four major domains using the Onion Model. From the outer layer to the inner layer were knowledge and skills, professional abilities, professional quality, and personal traits. The qualitative data revealed several novel competencies, including triage knowledge, self and mutual medical aid, outdoor survival skills, and sense of discipline.

**Conclusions:**

The application of the Onion Model promotes the understanding of competency and strengthens the theoretical foundations of this study. New competencies can enrich the content of the competencies of OR nurses. The results of this study can be used for clinical recruitment, evaluation and training of OR nurses in mobile surgical teams. This study encourages further research to develop competency assessment tools and training programs for OR nurses.

## Background

Competency was first proposed by McLeland, which is defined as a personal trait or a group of habits, leading to more effective or better work performance. Competency is composed primarily of five components: knowledge, skills, self-concept and values, motives, and traits [1]. American psychologists define competency as the personal characteristics that distinguish superior performers from mediocre performers in a particular environment. It can be knowledge, skill, social role, self-image, trait, motive, etc. [2]. The Association of Operating Room Nurses (AORN) defines the competency of an operating room (OR) nurse as the knowledge, skills, and abilities required to perform the professional functions of a registered nurse (RN) in the OR [3]. In China, the definition of OR nurse competency is similar to the above [4].

In recent years, with the frequent occurrence of public health emergencies, conflicts and natural disasters worldwide, various forms of medical rescue teams are playing an increasingly prominent role. The mobile surgical team is a special team. In the turbulent and severe disaster rescue environment, mobile surgical teams can quickly rescue the wounded and perform simple operations (advanced life support, damage control surgery, damage control resuscitation), which can shorten the rescue time, reduce mortality and disability [5, 6]. Mobile surgical teams can be deployed independently or as part of a mobile medical team or field hospital. They can provide advanced care with sufficient flexibility and versatility, and they are more convenient and suitable for surgery than field hospitals [7]. So more and more countries and hospitals establish mobile surgery teams, including International Committee Of Red Cross (ICRC) mobile surgical teams [8], U.S. Navy Fleet Surgical Team (FST) [9], U.S. Army Forward Surgical Teams (FSTs) [10], Army Forward Resuscitative Surgical Teams (FRST) [11], French Forward Surgical Teams (FSTs), French Surgical Life-saving Module (SLM) [12], Emergency medical rescue surgical team, China Naval forward surgical team (NFST) [13].

OR nurse in the mobile surgical team plays a unique role. In China, the mobile surgical team is mainly composed of OR nurses, surgeons, and anesthesiologists [14]. OR nurses in the surgical team comprise two roles: scrub (or instrument) nurse and circulating (or floor) nurse. The scrub (or instrument) nurse assists the surgeon, administrates the equipment, and drapes the patient. The circulating (or floor) nurse serves the surgical team, positions and disinfects the patient, manages activities outside the sterile field, supervises nursing care during the procedure and acts as the patient’s advocate [15]. In China, an OR nurse needs to have the competencies of scrub nurse and circulation nurse to be able to perform either role at any time.

The competency of OR nurses is one of the most crucial indicators of patient safety and professional practice standards [16, 17]. They are essential in the delivery of nursing care to meet the varying needs of injured patients, surgeons, anesthesiologists, and the demands of changing environments and circumstances. In order to maintain and improve a variety of competencies, they participate in surgery work in peacetime just like OR nurses in ordinary hospitals. When deployed, their work sites include battlefields, surgical tents, surgical vehicles, surgical cabins, aeroplanes, ships, or field hospitals [18]. They need to care for patients with unusual injuries, including multiple injuries, puncture wounds, spinal cord injuries, traumatic amputations, crush injuries, head injuries, burns, infectious diseases, etc. [19–21]. Different working environments, regions, service objects, etc., lead to the unique characteristics of the work. Therefore, OR nurses in mobile surgical teams have unique professional skills and comprehensive abilities that differ from OR nurses in ordinary hospitals.

Competency models have been used in job analysis, performance management, and human resources training and development [22]. The American academic Boyatzis’ Onion Model, which has multiple interconnected layers and is an enriched competency model [23]. The outer layer of the Onion Model contains knowledge and skills that can be easily seen and cultivated. The middle layer includes self-concepts and values. The core of the Onion Model encompasses traits and motivations that are difficult to assess, making training in this area also difficult [2]. Boyatzis’ Onion Model can offer a thorough viewpoint on exploring competencies and competency-based nursing education [23].

To our knowledge, many studies have reported on the competencies of OR nurses in hospitals. Few studies have explored the competencies of OR nurses in mobile surgical teams. The Onion Model can better explain the competencies of the OR nurses in the mobile surgical team at different levels. Qualitative research approaches can uncover deeper meanings of human experiences and behaviors [24]. This research method can help us to deeply and extensively explore the competency of OR nurses in mobile surgical teams. In order to gain a better understanding of OR nurses’ competencies, we conducted a qualitative study on the theoretical basis of the Onion Model, which can provide enriched theoretical guidance for competency-based nursing education and competency building.

## Methods

### Design

We conducted semi-structured interviews with surgical team members using the qualitative descriptive design. Our research team has extensive experience in qualitative research. NAF has more than 10 years of experience in OR nursing and mobile surgical teams, CZ and ZXL have about 10 years of experience in medical team management and training, and MHJ and LY have extensive experience in nursing education.

### Participants

Based on the requirements of qualitative studies, we used purposive sampling method to obtain a small convenient sample at some convenient institutions [28]. Surgical team members were selected based on the following criteria. Inclusion criteria: (1) participation in the mobile surgical team for at least three years; (2) performing rescue missions (public health emergency, conflict or natural disaster) as a member of the surgical team; (3) voluntary participation in the study. Exclusion criteria: no longer in a mobile surgical team when the interview was conducted. Before the investigation, each participant signed an informed consent form. When no more new themes appear in the inductive content, the saturation is reached. In total, we interviewed 21 surgical team members.

### Data collection

The Onion Model and the framework for behavioural event interviews served as the basis for the interview outline [1, 2]. The interview outline includes the following questions, of which both doctors and nurses answered: (1) background enquiries, such as gender, age, profession, education, job title, years of work, and years of participation in the medical team; (2) What competencies do OR nurses in the mobile surgical team need? The question only doctors answered: What impressed you about the OR nurses during the deployment? Questions that only the nurse answered: (1) What made you feel more deeply during the deployment? (2) What were the most successful and unsuccessful events you had as a mobile surgical team member during deployment?

From April to May, 2022, after obtaining institutional permission and the names and contact information of surgical team members, the research team contacted them via telephone or WeChat (a popular Chinese social media app), introduced study objectives, and asked whether they would be interested in participating. After explaining the study’s significance and anonymity to willing interview subjects from 10 mobile surgical teams, they were informed that they could withdraw at any time. A formal interview was organized at a time that convenient for the participants. To ensure that the whole interview process was completed smoothly, we chose a quiet, relaxed and undisturbed place. During the interview, the researcher confirmed the unclear content of the interviewee through retelling and rhetorical questioning. With the consent of the interviewees, we recorded the whole process of the interview with a recording pen. The Mandarin -speaking interviews were conducted, digitally recorded, and then submitted to a site that provides online transcription. Each interview was recorded and lasted roughly 30 min.

### Data analysis

The data were analysed using the Mayring’s qualitative content analysis method which includes deductive and inductive steps [25]. Within 24 h of each interview, two authors independently listened to the recordings and checked them verbatim in Microsoft Word. In the induction step, the research team read the text several times for a general understanding and then outlined the meaning of units and phrases that were significant to the research purpose and question in Excel. Every meaning unit was distinguished, summarized, condensed and labelled with a code and subsequently classified into categories and subcategories based on similarities. A deductive approach was used to relate the categories and subcategories to the three layers of the Onion Model. The categories and subcategories were determined once all transcripts were inducted and deduced. Before agreeing to the final result, the project team resolved any discrepancies in interpretation using a consensus-building process. Coding and content analysis was carried out in Chinese. The categories, subcategories and corresponding quotes in the manuscript were translated into English and proof-read by a professional translator.

### Ethical considerations

The Medical Ethics Committee of Chongqing Army Medical University received the study protocol, authorized the study, and declared an exemption. Participants received assurances of confidentiality and anonymity. They were made aware that participating was completely optional and that doing so would have no adverse effects. Before taking part in this study, each participant signed the consent form. The informants are not identified in the article because their identifying details were removed after the interviews were transcribed.

### Rigour

Rigour was ensured using Guba and Lincoln’s criteria for credibility, dependability, confirmability, and transferability [26]. The credibility of this study was achieved via member checks and field notes [27, 28]. The two researchers had extensive experience in qualitative research methods and had no relationship with the participants, which increased the credibility of the study. This study was directly cited by appropriate numbering (e.g., P01) and the results were compared with previous literature to ensure dependability. Two experienced OR nurses discussed the results to assess the dependability of the findings. Confirmability was established by documenting and reporting all steps of the study. We have provided detailed descriptions of quotes with the findings to ensure transferability.

## Results

### Participant details

The final sample was made up of 6 male and 15 female participants from 10 mobile surgical teams in China. Besides, fifteen OR nurses, four surgeons and two anesthesiologists. The average age was 36.00 ± 5.92 years (range: 27–47), the average working time was 14.43 ± 7.24 years (range: 4–27), and the average years of participating in the medical team was 8.67 ± 5.10 years (range: 3–20). Sixteen individuals had bachelor’s degrees, while five participants had master’s or doctoral degrees. Table 1 includes a list of the participants’ specific demographic details.

**Table 1:** Demographic characteristics of participants

Variable	n	%
Gender		
Female	15	71.4
Male	6	28.6
Profession		
OR nurse	15	71.4
Surgeon	4	19.1
Anesthesiologist	2	9.5
Age(year)		
≤30	6	28.6
30–40	10	47.6
≥41	5	23.8
Current Level of Education		
Bachelors	16	76.2
Masters	3	14.3
Doctoral	2	9.5
Job Title		
Primary	5	23.8
Intermediate	11	52.4
Senior	5	23.8
Working years		
≤ 10	7	33.3
10–20	10	47.6
≥ 21	4	19.1
Participation in the surgical team (year)		
≤ 5	7	33.3
5–10	9	42.9
≥ 11	5	23.8
Participation in rescue missions (month)		
≤ 10	8	38.1
10–20	5	23.8
≥ 21	8	38.1

### Competencies of OR nurse

**Fig. 1:**
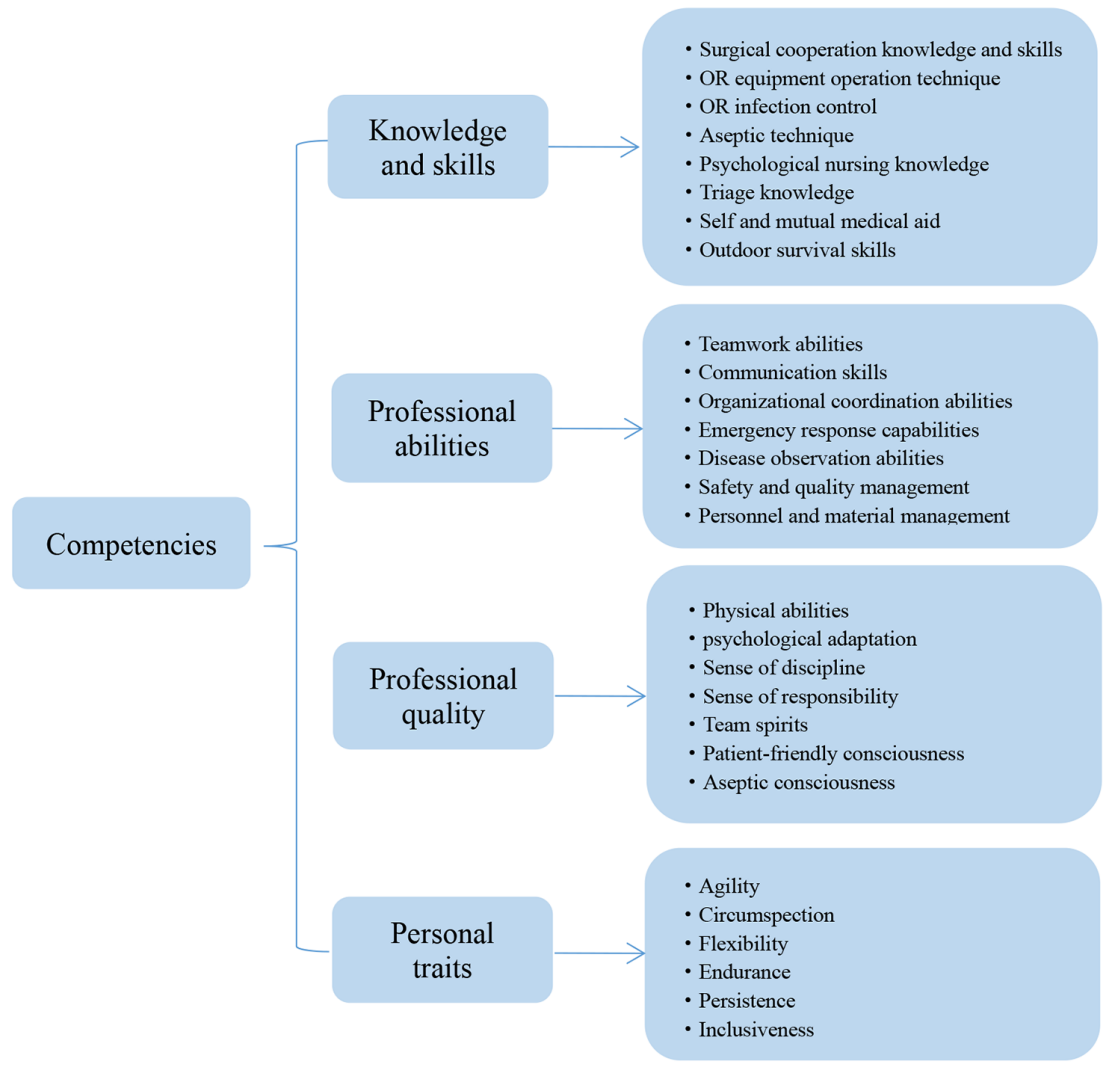
The competencies of OR nurses in mobile surgical teams

**Fig. 2:**
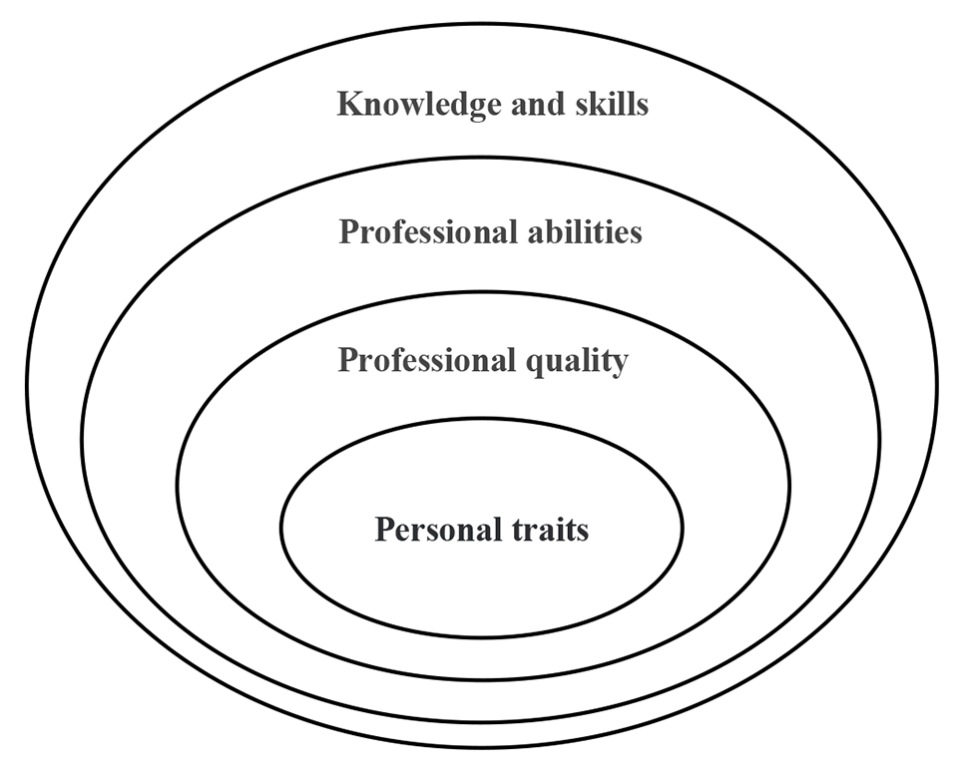
The onion competency model of OR nurses in mobile surgical teams

Twenty-eight competencies were identified from data analysis and grouped into four key domains, including knowledge and skills, professional abilities, professional quality, and personal traits (Fig. 1). Knowledge skills and professional abilities are equivalent to the outermost layer of the Onion Model, which is divided into two layers in this study. Professional quality is equivalent to the middle layer of the Onion Model, namely the self concept, attitudes, and values. Personal traits are equivalent to the traits and motives of the Onion Model. As illustrated in the onion competency model of OR nurses in mobile surgical teams (Fig. 2).

#### Knowledge and skills

Knowledge and skills make up the onion competency model’s outer layer. The majority of participants said that knowledge and skills were the most basic requirements for becoming an OR nurse, and also the basis of competency level. OR nurses in mobile surgical teams in China may encounter two types of nursing contexts including the daily routine surgical care and field medical rescue care. Therefore, they need to have special knowledge and skills in addition to conventional surgical care when performing rescue missions.

Most of the participants expressed that OR nurses need to be proficient in surgical cooperation knowledge and skills, OR equipment operation technique, OR infection control, aseptic technique, and psychological nursing knowledge. Psychological nursing knowledge is essential, especially in the face of patients with post-traumatic stress disorder.

*As a surgeon, I think a good scrub nurse works well with them. For example, during the operation, if I need something, they can provide it to me quickly… Better instrument nurses even know the surgical procedures and habits of the surgeon, and know what instruments and supplies are needed for each step (P13)*.

*The instruments and equipment used in field surgery differ from those used in hospital ORs, such as field operating table, field electrotome, and field aspirator. The operation and use of these instruments and equipment must be learned, trained, and proficient before deployment tasks (P03)*.

*Infection control in the OR under field conditions is a weak point, because the airtight conditions in the OR under field conditions are relatively poor and limited… In addition, some patients have not been fully prepared before the surgery, so infection control is highly required during the operation, which is a challenge for the OR nurses. At the same time, surgery will generate a lot of waste, and the disposal and placement of medical waste is also a problem, I think (P12)*.

*I think the OR nurses should learn some psychological knowledge, pay attention to the psychological condition of the wounded, and carry out psychological nursing work such as comfort and soothe when necessary (P17)*.

Performing rescue missions in the field, OR nurses also needed to master triage knowledge, self and mutual medical aid, and outdoor survival skills. These are unique and novel competencies.

*In the field environment, due to limited conditions or critical condition of the wounded, many examinations and tests are insufficient when the wounded enters the OR. The nurses and doctors in the OR should first have a triage on the wounded then decide what surgery to do, which part should be operated on first, and which part should be done later. Wrong triage will delay the condition, so I think triage is essential (P01)*.

*OR nurses also need to be equipped with outdoor survival skills. When treating the wounded in the field, they should protect themselves first, and then treat the wounded (P02)*.

#### Professional abilities

The second layer is professional ability. Professional ability refers to the ability that OR nurses should have when performing routine surgical work and deployment tasks, mainly including seven competency indicators. The OR environment is flexible and changeable, and the deployment environment is even more arduous and dangerous. The condition of trauma patients is usually urgent, severe, complex and so on. These factors pose challenges to the professional abilities of OR nurses in the mobile surgery teams.

OR nurses play dual roles as team members and nurses. As surgical team members, the OR nurses in this study emphasised the importance of teamwork abilities, communication skills, and organizational coordination abilities. Effective communication, according to interviewees, was crucial for maximizing OR cooperation. In addition, ensuring good information, wounded, and supply flow within the entire team was critical.

*We are a team, and I think teamwork is essential. As a team collaborator, part of the work should be undertaken by the OR nurse… We should clarify our responsibilities and tasks clearly, and cooperate in the teamwork process (P02)*.

*I think the coordination ability of an OR nurse should be better. If she is a circulating nurse, she has to coordinate the operating room, the wounded, the surgeon, etc… I think coordination ability is quite important.(P12)*.

*I think the communication ability of OR nurses is vital, especially with doctors, which is crucial for the smooth operation. When our surgical team encounters problems, doctors also ask the operating room nurse for advice and solve problems together through communication (P15)*.

In the constantly changing working environment and the condition of the wounded, some participants believed that the OR nurse must be able to detect in time, make the rapid and appropriate judgments, analysis and respond, and be able to mobilize personnel and material resources to solve the problem fully. Even the most routine procedures can alter in a split second, so OR nurses have to monitor them all the time and be able to change with them to make sure the process is safe.

*As an anesthesiologist, I think the OR nurses most need to have the ability to adapt and respond to emergencies. Because we perform operations in the wild, there are many unexpected situations, so it is imperative to find, deal with and cooperate with what happens quickly (P03)*.

*If the wounded is under conditional monitoring, the OR nurse must pay attention to the monitor at any time. If the monitoring conditions are not met, you may need to judge from the patient’s complexion, lips and face, etc… You must be able to judge hemorrhagic shock, coma, confusion and other states (P08)*.

*I think the management of supplies is also vital. You should be familiar with all the materials. You need to know where the supplies are, so that you won’t be particularly busy when treating the wounded (P09)*.

*To ensure the safety of the operation and improve the quality of the operation, the OR nurses need to monitor whether the aseptic operation of the surgeon is in place, and the supervision should be strong. Our nurses are still not in place in this aspect… At the same time, the OR nurse must manage the number of surgical instruments (P06)*.

#### Professional quality

The third layer is professional quality as a member of the mobile surgical team. Professional quality is the code of conduct that OR nurses should observe when performing rescue missions. They need to have unique professional qualities. The study’s participants emphasized the value and significance of physical abilities, psychological adaptation, and sense of discipline, when OR nurses performed the deployment rescue missions.

*When performing deployment* missions, *especially in harsh environments, if the OR nurse’s physical ability is not good, it is difficult to competent for the job. Moreover, the operation often requires nurses to work around the clock without rest, so the physical ability requirements are relatively high.(P21)*.

*In the field environment, there will be many emergencies. As an OR nurse, she needs good psychological adaptation to deal with them calmly. (P05)*

*I think it’s important to have a sense of discipline during the deployment, to follow the rules and regulations of the team and the medical.(P16)*

When working as an ordinary OR nurse, the professional qualities they need include a sense of responsibility, team spirits, patient-friendly consciousness, and aseptic consciousness in our study.

*When we carry out deployment tasks, we carry a lot of supplies, which is quite messy. Nurses can take responsibility for the supplies and worry about them, and do their best (P09)*.

*I feel that the team members cooperate, I can’t only care about my own business, I don’t care about the work of other members, I feel that we are a whole, even if it is not my work, if I can do it and I have time, I’ll help. It’s a team (P02)*.

*I think the nurses can comfort the wounded when necessary, which can give them enough sense of security… I think the patient-friendly consciousness of nurses should be strengthened (P09)*.

#### Personal traits

Personal traits are the innermost layer of the competency model of the OR nurses in the mobile surgical team. Personal traits are the key, unique, and common personal characteristics that OR nurses should show when performing rescue missions. The traits found in this study included agility, circumspection, flexibility, endurance, persistence, and inclusiveness. Rescue missions are often emergent requiring OR nurses to have the characteristics of agility, circumspection, flexibility. Moreover, the rescue work is heavy and laborious that require patient, persistent, and tolerant OR nurses.

*I think our OR nurses should be quick and agile, for example, they can quickly take out what I want during the operation (P11)*.

*Although the work in the OR is trivial, everything is significant. If you are not careful, it will threaten the lives of patients… the OR nurses need to be very careful and thoughtful when working (P02)*.

*There are many essential differences between performing rescue missions in the field and working in the hospital. It is more difficult. In addition, there are many trivial things in the OR, which further increases the workload of the OR nurses. It requires the OR nurses to have enough perseverance and can withstand hard work (P18)*.

*The environment of fieldwork is not good… You can’t complain. You have to face the difficulties deep in your heart. At the same time, you should be tolerant of your team members, because many team members spend less time with each other and are not familiar with each other, which will inevitably lead to friction (P17)*.

## Discussion

The competencies of OR nurses are conceived as the relationship between technical and non-technical skills [17, 29]. In our study, technical skills consisted of knowledge and skills, and non-technical skills included professional ability, professional quality, and personal traits. Additionally, the Onion Model’s division of various elements into four layers gives this study a solid theoretical foundation, promotes a clear understanding of competency, and aids in competency-based nursing education and training.

The outermost layer of the onion competency model is technical skills, that is, knowledge and skills that can be easily seen and cultivated. As specialist nurses, the knowledge and skills of the OR nurses are the basis and necessary conditions for all work. The technical areas include operating room-specific nursing knowledge and skills [30]. In Sweden, specialist OR nurses are responsible for surgical tools, instruments, equipment, aseptic techniques, infection and complication prevention, and biological specimen handling [16, 31]. Surgical team members are considered accountable for adhering to asepsis guidelines and patient safety concerns in the OR [32]. In the OR, controlling asepsis and hygiene is crucial for preventing postoperative wound infections [33]. Wars, disasters, terrors, conflicts and other traumatic life events are unpredictable and often sudden events that cause great damage, destruction and human suffering. Psychological effects can be harmful and far-reaching [34]. Psychological nursing knowledge is important and should not be ignored. Shin and Kim found that OR nurses had the highest educational demand for knowledge and skills to improve competency [35].

For the OR nurses of the mobile surgery team, this study found that they also need to acquire unique knowledge and skills, including triage knowledge, self and mutual medical aid, and outdoor survival skills, which are necessary for OR nurses to master when they go out of the fixed hospital and perform rescue missions in the field. Triage is a crucial nursing skill for managing emergencies [36]. The purpose of triage is to identify the severity of the injury and reduce the negative consequences through rapid evaluation. Triage decisions are often made quickly, independently, and under time constraints, so they can have a serious impact on patient outcomes and flow [37]. Triage knowledge and professional abilities play important part in nurses’ decision-making. The first aid timeliness rule reveals that self-help and mutual aid have the highest first aid timeliness value (the highest success rate). Nurses should not only rescue the wounded, but also be able to save themselves if they are in danger. Nurses in large hospitals have less exposure to the wild, so they are less able to cope with nature and survive in harsh environments [38]. Mobile surgical team nurses need outdoor survival skills so that they can better cope with severe situations where disaster, terror, conflict and even war occur frequently. These unique knowledge and skills enrich the competency content of OR nurses, and are also the focus and challenge of the future training and education of mobile surgical team nurses.

The mobile surgical team in China is mainly composed of surgeons, anaesthetists, and OR nurses, to provide the best care for the wounded and safe surgical care. OR nurses being the largest professional group represented play a variety of roles when working with professionals on the surgical team. They collaborate with surgeons, anesthesiologists and nurses to promote surgical performance and increase the likelihood of good patient outcomes [39]. When an emergency occurs, a mobile surgical team is selected temporarily, and the team members are often not familiar with each other. Patient safety may be compromised if there is a lack of continuity and stability in team familiarity, which could lower individual and team performance [40]. For such a team, communication skills, teamwork skills, and coordination abilities are crucial. Studies have found that communication in the OR has a considerable effect on the performance of the surgical team, and thus has a significant impact on patient safety and treatment outcomes [41]. Communication skills, as a non-technical skill, complement technical skills to handle emergency deployment tasks efficiently and safely. Furthermore, effective communication can help wounded feel less anxious. Teamwork skills in the surgical team are associated with a reduction in adverse events, as well as improved patient outcomes, including reductions in mortality and complications [42]. Good cooperation (i.e., team-based attitudes), communication (i.e., the exchange of information between members), and coordination (i.e., teamwork behaviours) among surgical team members create a tranquil workplace for the workforce, reducing medical errors and complications for patients [39, 43].

The OR is a complex, dynamic, and stressful work environment [44], especially when dealing with emergency rescue deployments. In this setting, life-threatening circumstances and occurrences needing quick decisions frequently occur. Surgical team members need more than specialized knowledge and technical skills. To achieve safe, effective patient outcomes, OR teams must comprehend the complexity of clinical circumstances, coordinate patient care utilizing modern tools and procedural approaches while reacting to frequently quick patient conditions changes [44]. von Vogelsang et al. found that OR nurses need to be able to synthesize knowledge and analyze, evaluate, and handle complex situations [16]. Moreover, they should have the competencies common to other specialized nurses, such as disease observation ability, emergency response capability, and management ability, which are consistent with the research results of Wang et al. [14]. To maintain wounded safety, infection prevention, psychological safety, environmental safety, and team safety, the OR nurse is responsible for the WHO checklist’s needle, sponge, and tool count as well as the management of specimens [45–47]. OR nurses must know where everything is and monitor everything in the OR. Additionally, the OR nurse is in charge of directing traffic inside the OR [48]. A scarcity of resources might occur during deployment for an OR nurse, thus managing supplies and adapting are crucial skills [49]. The ability of an OR nurse also includes dispute resolution, resource organization, and prioritization in light of the constantly shifting and unpredictable needs of the surgical setting [29]. The professional abilities excavated in this study mainly focus on the abilities required for team work, and provide a basis for the cultivation and formation of professional abilities of OR nurses in the future.

Quality is an essential quality of physical and mental development, which is relatively stable once formed. Professional quality is a comprehensive reflection of labourers’ understanding and adaptability to social occupation. It is the third layer of the onion competency model of OR nurses, which is not easy to find and cultivate. In our study, the professional quality of OR nurses includes physical abilities, psychological adaptation, sense of discipline, sense of responsibility, team spirit, patient-friendly consciousness, and aseptic consciousness. The OR has a large workload, high work intensity, high work pressure [44, 50], irregular working hours, changing and fast-paced environment, unexpected situations, and uncertain demands [29]. In addition, the mobile surgical team often face urgent rescue missions, which puts forward higher requirements on physical ability and psychological adaptation. Physical and mental health are fundamental conditions for working. And the professional attitude or quality of an OR nurse requires concern for others, service consciousness, a sense of responsibility, and team spirit [51, 52]. During surgery, OR nurses ensure that the wounded receive perioperative care that is person-centred [53]. Wang et al. study shows that OR nurses need to possess the essential competency characteristics of patient-friendly consciousness [14]. Janatolmakan and Khatony found that nurses who lack teamwork spirit can lead missed nursing care [54]. In our study, a sense of discipline is a particular competency. During deployment, a sense of discipline is essential for a team to ensure safety and completion of the mission. Although this part of the competencies is not easy to develop, they can help managers develop long-term training programs and appropriate educational methods to develop OR nurses’ competencies. At the same time, they provide a basis for managers to select suitable OR nurses for mobile surgical teams.

Personal traits are the innermost layer of the onion competency model of the OR nurses. They are the inner, difficult to measure, difficult to find and change aspects of people. Previous studies found that OR nurses have personal characteristics of agility, attention to detail, stress resistance, flexibility, endurance, persistence, and inclusiveness [51, 52, 55, 56], consistent with our findings. With an operations tempo, mobile surgical teams must be small and agile [57]. To rapidly locate any surgical supplies or medications, OR nurses must be agile and quick-thinking, especially in challenging or unexpected situations. This calls on them to exercise caution, to pay attention to both the big picture and the minute particulars, and to always be aware of the needs of the patient, the equipment, and the surgical team. While listening to the surgeon, the OR nurses can help with the process while keeping an eye on the patient, the equipment gauges, and other signs that everything is proceeding according to plan. Most surgical teams are set up ad hoc and may consist of different team members for each deployment. Team members may be unfamiliar with each other’s skills, abilities, work habits and styles. Lack of understanding of each further increases the likelihood of miscommunication and disruption during surgery [40]. These conditions challenge the adaptive capacity, flexibility, and inclusiveness of team members. Higher and more modern standards are being set for nursing talent in quantity, quality, and structure as OR nursing continues to advance. Exploring personal traits will help managers select suitable and competent OR nurses for the mobile surgical team.

The study has its limitations. First, the deductive approach to data analysis and the usage of the Onion Model as a theoretical framework for interview outlines may introduce some biases. Second, the results are time- and context-specific, so they may not be applied to all OR nurses. Third, due to the limited experience of interviewees in different deployment tasks, the results will be biased. The last, interviews were conducted in Chinese, then analyzed and translated into English. Although the authors worked hard to ensure proper translation with the help of professional English editors, there was still a small risk that the process could affect the results of the study.

## Conclusions

This study explored the competencies of OR nurses in mobile surgical teams through qualitative research. A variety of novel competencies were discovered when investigating the OR nurses. The application of the Onion Model, which also promotes knowledge of competency, strengthens the theoretical foundations of this study. The results of this study enrich the contents of OR nurses’ competency, and suggest that the competency of OR nurses is not only having a wealth of theoretical knowledge and specialized technical abilities, but also the complementary results of various competency characteristics. Therefore, it is recommended that managers incorporate the professional abilities, professional quality and personality traits of specialist nurses into the recruitment, assessment or training criteria for OR nurses of mobile surgical teams. Future research is required to develop reliable, professional training programs and scientific competency assessment tools for OR nurses in mobile surgical teams. It is also necessary to research how to help mobile surgical teams’ OR nurses adjust to deployment to support the growth of a skilled, adaptable, and ready nursing workforce. So as to impact on the safety of surgery-related patient, improve the performance of OR nurses, and reduce the mortality and disability of trauma.

## Data Availability

The datasets used and/or analysed during the current study are available from the corresponding author on reasonable request.
